# Inequalities in Stroke Patients' Management in English Public Hospitals: A Survey on 200,000 Patients

**DOI:** 10.1371/journal.pone.0017219

**Published:** 2011-03-02

**Authors:** Antonio Ivan Lazzarino, William Palmer, Alex Bottle, Paul Aylin

**Affiliations:** Department of Primary Care and Public Health, Imperial College London, London, United Kingdom; Julius-Maximilians-Universität Würzburg, Germany

## Abstract

**Background:**

According to clinical guidelines, every patient affected by stroke should be given a brain-imaging scan (BIS) - Computerized Tomography or Magnetic Resonance Imaging - immediately after being admitted to hospital.

**Aim of the study:**

To describe the variation in use of BIS among English public hospitals and identify any patient groups being excluded from appropriate care.

**Methods:**

We collected hospital administrative data for all patients admitted to any English public hospital with a principal diagnosis of stroke from 2006 to 2009. We calculated the proportion of patients treated with BIS in the whole sample and after stratification by hospital. We compared hospitals' performance using funnel plots. We performed a multiple logistic regression analysis using BIS as outcome and age, gender, socio-economic deprivation, and comorbidity as covariates.

**Results:**

In English public hospitals there are about 70,000 emergency admissions for stroke per year. Nationally, only 35% receive a BIS immediately, and only 84% receive it within the admission. There is large variation in the use of BIS for stroke patients among English public hospitals, with some of them approaching the recommended 100% and some having very low rates. Young (P<0.001), male (P = 0.012), and least socio-economically deprived patients (P = 0.001), as well as patients with fewer comorbidities (P<0.001) appear to have more chance of being selected for a brain scan.

**Conclusion:**

Some English public hospitals appear to be falling well below the clinical guideline standards for scanning stroke patients and inappropriate patient selection criteria may be being applied, leading to health inequalities.

## Introduction

Stroke is the leading cause of adult disability in the United States and Europe. Worldwide, it is the number two cause of death and may soon become the leading cause [1]. In England, there are approximately 70,000 emergency admissions for stroke each year and it is third largest cause of death in England and single largest cause of adult disability. Stroke costs the UK economy about £8 billion a year, including £3 billion in direct costs to the NHS [2].

Stroke can be classified into two major categories: ischaemic and haemorrhagic. Ischemia is due to an interruption of the blood supply, while haemorrhage is due to rupture of a blood vessel or an abnormal vascular structure. 80% of stroke is due to ischaemia; the remainder is due to haemorrhage. Some haemorrhages develop inside areas of ischemia (“haemorrhagic transformation”). It is unknown how many haemorrhages actually start off as ischaemic stroke [3].

The sub-classification of stroke into the two categories, ischaemic or haemorrhagic, is crucial for the treatment choice. Ischemic stroke is caused by a thrombus (blood clot) occluding blood flow to an artery supplying the brain. Definitive therapy is aimed at removing the blockage by breaking the clot (thrombolysis) or by removing it mechanically (thrombectomy). Patients with haemorrhagic stroke require neurosurgical evaluation to detect and treat the cause of the bleeding, although many may not need surgery. The thrombolytic treatment, key in treating ischemic stroke, can make bleeding worse and cannot be used in haemorrhagic stroke.

Imaging techniques are essential tools for the correct diagnosis and sub-classification of stroke and therefore for the treatment choice. The current international guidelines for the management of patients with suspected acute stroke strongly recommend the use of either computerised tomography (CT) or magnetic resonance imaging (MRI) for all patients, and studies have been implemented to compare the performance of those two techniques [4]–[6]. The National Stroke Strategy in England suggested that “patients be scanned in the next slot within usual working hours, and within 60 minutes of request out-of-hours”, and presented the proportions of patients scanned within one hour and within 24 hours as measures of success [7].

The aim of this study was to describe the variation in the use of brain-imaging techniques for the management of patients affected by stroke in English acute hospital trusts and identify any patient groups being excluded from appropriate care, using routinely collected administrative data.

## Methods

### Hospital Episode Statistics (HES) Data

The HES database comprises data gathered locally through Patient Administration Systems or the Hospital Information System, which contains clinical and administrative information on all admissions to NHS hospitals in England since 1986. The basic unit of measurement in HES is the Finished Consultant Episode (FCE), defined as the period of time during which the patient is under the care of a consultant until they are either transferred to another consultant or discharged. Procedures are classified according to the Office of Population Censuses and Surveys Classification of Surgical Operations and Procedures (4^th^ revision) (OPCS-4) and diagnostic coding including primary and secondary coding is recorded according to the International Classification of Disease (10^th^ revision) (ICD-10).

Secondary diagnosis codes can be used to create the Charlson comorbidity index [8], which includes diabetes mellitus, cancers, cerebro-vascular disease, liver disease, kidney disease and other factors. Moreover, the number of emergency admissions in the previous twelve months can be calculated for each admission and can be considered as a proxy for patient's comorbidity level and disease severity. The Carstairs index of deprivation is a geographically-based deprivation score that is based on four census indicators (low social class, lack of car ownership, overcrowding and male unemployment). Assuming that patients' socio-economic status can be estimated by the deprivation score of the area where they live, this index can be used in epidemiological studies to adjust for patients' socio-economic deprivation. We linked HES data and Carstairs index for all patients according to their home postcode [9] and assigned each patient to a score to estimate their socio-economic status. The database also contains variables such as age and gender that can be used as covariates.

### Ethics

Written consent by the patients for their information to be stored in the hospital database and used for research was not needed because have approval under Section 251 (formerly Section 60) granted by the National Information Governance Board for Health and Social Care (formerly the Patient Information Advisory Group (now the NIGB) to provide measures of healthcare quality by provider. We also have approval from the South East Research Ethics Committee to carry out this research.

### Analysis

We examined HES data for the period 1 April 2006 to 31 March 2009 (financial years 2006/07 to 2008/09) for all NHS non-specialist acute trusts in England. All patients admitted as an emergency with a primary diagnosis of stroke were selected using the ICD-10 codes I61, I63 and I64 and admission codes 21 to 28 (emergency admission). Brain-imaging procedures were identified using the OPCS-4 codes U05 and U21. The extracted data were cleaned, which included removing duplicate FCEs. The valid FCEs were linked together into admissions, and admissions were linked together if the patient was transferred to another hospital. We excluded those patients who died on the same day of the admission. We also excluded patients younger than 17 years old.

We have considered three outcomes: 1) brain scan performance during the same day of admission (“same-day scan”); 2) brain scan performance during the same day of admission or the day after (“one-day scan”); 3) brain scan performance anytime during the hospitalisation (“any-time scan”).

For each outcome we calculated the proportion of patients treated within these timeframes by year and then plotted the 2008-09 rates of all hospital trust separately using funnel plots with 99.8% control limits to identify statistical outliers. In a funnel plot each trust is represented by one dot and the dot's position inside the plot is given by the total admissions (x axis) and the proportion of admissions in which the patients was tested with a brain scan (y axis). The plot also contains the average proportion of admissions in which the patients were tested with a brain scan (horizontal line) and associated control limits (the two curves that form the typical funnel shape). If very few trusts lie outside the funnel (by chance about 0.2%, or none of our 150 trusts, are expected to lie outside the 99.8% confidence intervals) the performance among hospital trusts can be regarded as being consistent with purely random variation. Conversely, if many trusts lie outside the funnel then the brain scan performance can be regarded as being heterogeneous, i.e. there is greater than expected variation in performance among trusts [10].

Subsequently we studied the chances of receiving a brain scan as a function of the available covariates: age, gender, socio-economic status (Carstairs), comorbidity (Charlson), and number of emergency admission in the previous twelve months. We fitted a multiple logistic regression model on the 2008-09 data to estimate the odds of receiving a brain scan any time in the hospitalisation. Then, to assess the urgency with which the scan is performed, we fitted another multiple logistic regression model on the 2008-09 data to estimate the odds of a patient receiving a brain scan on the same day of admission, restricting to those that had a scan at any time during hospitalisation. All available covariates have been considered as potential risk factors and have been included in the multiple models using the forward stepwise approach, i.e. the variables were sequentially added to an “empty” (intercept only) model one at a time giving priority to those that had shown the strongest evidence of association at the univariate stage (smallest P value). At each round the importance of the added variable was assessed according to changes in the sum of squares, odds ratios and P values (cut off  =  0.05) of all variables in the model. When we found some non-linear relationship between covariate and outcome we categorised the covariate.

## Results

In England there were 209,174 emergency admissions for stroke to NHS non-specialist hospitals in financial years 2006/07, 2007/08, and 2008/09. The proportion of patients recorded as having had a brain scan increased over time (Table 1).

**Table 1:** Description of brain scan performance for patients admitted as an emergency for stroke in all English public hospitals, excluding patients who died in the same day of admission.

Financial year	Total (1)	No. (%) of same-day scans (2)		No. (%) of one-day scans (3)		No. (%) of any-time scans (4)	
**2006/07**	69,014	9,796	(14.2)	18,196	(26.4)	30,392	(44.0)
**2007/08**	66,821	18,991	(28.4)	34,626	(51.8)	51,651	(77.3)
**2008/09**	73,339	25,452	(34.7)	43,267	(59.0)	61,798	(84.3)
**Total**	209,174	54,239	(25.9)	96,089	(45.9)	143,841	(68.8)

(1)No of pts admitted in emergency with a diagnosis of stroke.

(2)No (%) of patients tested with a brain scan during the same day of admission.

(3)No (%) of patients tested with a brain scan during the same day of admission or the day after.

(4)No (%) of patients tested with a brain scan anytime during the hospitalization.

**Figure 1:**
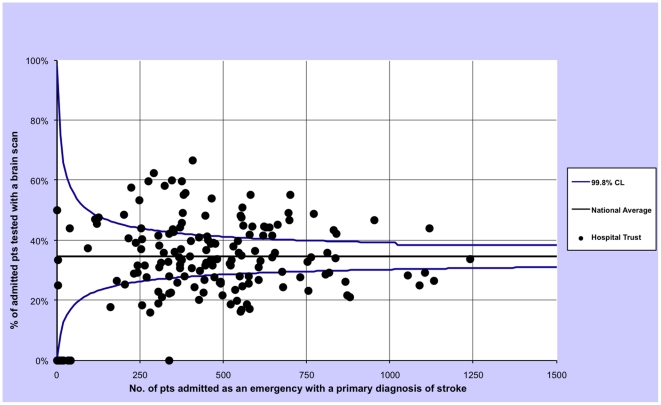
Funnel plot describing the variation in same-day scan performance for patients admitted in emergency for a stroke in all English public hospitals, excluding patients who died on the same day of admission, restricting to financial year 2008/09.

**Figure 2:**
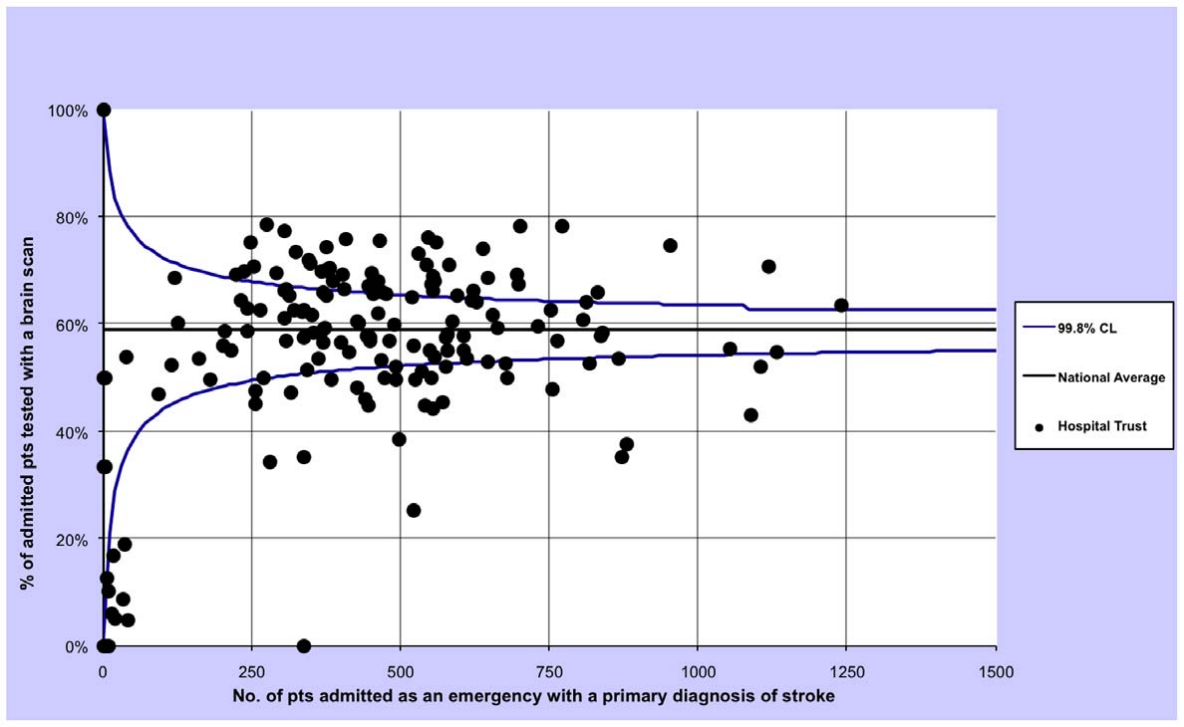
Funnel plot describing the variation in one-day scan performance for patients admitted as an emergency for a stroke in all English public hospitals, excluding patients who died on the same day of admission, restricting to financial year 2008/09.

**Figure 3:**
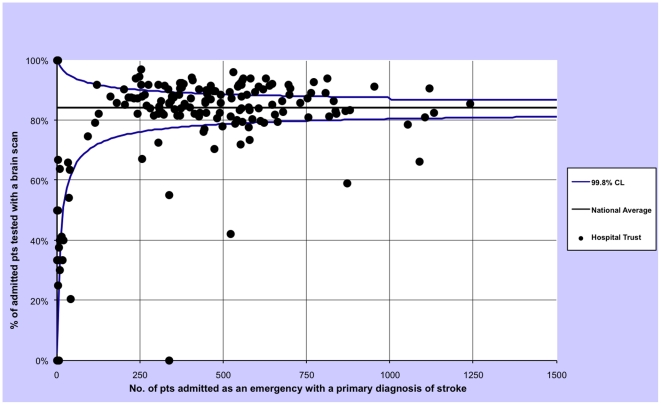
Funnel plot describing the variation in any-time scan performance for patients admitted as an emergency for a stroke in all English public hospitals, excluding patients who died on the same day of admission, restricted to financial year 2008/09.

The three funnel plots show an excess variation in brain scan performance among English public hospital trusts in financial year 2008/09, since more trusts than expected by chance lie outside the funnel (Figures 1, 2 and 3).

Table 2 shows the result of the logistic regression for the outcome any-time scan in financial year 2008/09. Age and gender appear to influence the probability of receiving a brain scan anytime during the hospitalisation. After having adjusted for all variables in the model male patients were more likely to be scanned than female patients, though the effect was small (OR = 1.05; P = 0.012; 95% CI = 1.01–1.10). Similarly, there was very strong evidence that young patients where more likely to be scanned than old patients (Adjusted OR for 10-year increase of age = 0.88; P<0.001; 95% CI = 0.87–0.90). Patients with higher comorbidity scores were more likely to be tested with a brain scan, whereas patients with more previous emergency admissions were less likely to be tested (Table 2).

**Table 2:** Multiple logistic regression for the odds of being tested with a brain scan at any time during the same emergency admission for stroke in public English hospitals, restricted to financial year 2008/09.

Factor	Category	Mutually adjusted OR	P	95% CI	
**Age**	10 year increase	0.88	<0.001	0.87	0.90
**Gender**	Female	1			
	Male	1.05	0.012	1.01	1.10
**Quintile of socio-economic deprivation (Carstairs)**	(least deprived) 1	1			
	2	0.98	0.555	0.92	1.05
	3	0.99	0.793	0.93	1.06
	4	0.99	0.735	0.93	1.06
	(most deprived) 5	0.97	0.432	0.91	1.04
	Not Known	0.43	<0.001	0.34	0.53
**Comorbidity score (Charlson)**	0	1			
	1	2.01	<0.001	1.84	2.20
	2	2.13	<0.001	1.94	2.35
	3	2.09	<0.001	1.87	2.32
	4	2.10	<0.001	1.84	2.41
	5	1.97	<0.001	1.62	2.41
	6+	2.28	<0.001	1.85	2.82
**Number of emergency admissions in the previous 12 months**	0	1			
	1	0.71	<0.001	0.68	0.75
	2	0.61	<0.001	0.56	0.65
	3	0.67	<0.001	0.61	0.74

Table 3 shows the result of the logistic regression for the outcome immediate scan in patients who had a scan during the hospitalisation, in financial year 2008/09. Age and gender appear to influence the speed with which the scan is performed. There was some evidence that after having adjusted for all variables in the model male patients were slightly more likely to be scanned quickly than female patients (Adjusted OR = 1.03; P = 0.067; 95% CI = 1.00–1.07). As for any-time scans, young patients were more likely to be scanned quickly than old patients (Adjusted OR for 10 year increase of age =  0.90; P<0.001; 95% CI = 0.89–0.91); the relations for comorbidity and number of previous emergency admissions were also similar (Table 3).

**Table 3:** Multiple logistic regression for the odds of being tested with a brain scan on the same day of admission in patients scanned at any time during the same hospitalisation, for emergency admission for stroke in public English hospitals, restricted to financial year 2008/09.

Factor	Category	Mutually adjusted OR	P	95% CI	
**Age**	10 year increase	0.90	<0.001	0.89	0.91
**Gender**	Female	1			
	Male	1.03	0.067	1.00	1.07
**Quintile of socio-economic deprivation (Carstairs)**	(least deprived) 1	1			
	2	0.95	0.048	0.90	1.00
	3	0.93	0.004	0.88	0.98
	4	0.91	0.001	0.86	0.96
	(most deprived) 5	0.94	0.024	0.89	0.99
	Not Known	1.44	0.001	1.16	1.79
**Comorbidity score (Charlson)**	0	1			
	1	3.41	<0.001	3.03	3.84
	2	2.98	<0.001	2.64	3.37
	3	2.99	<0.001	2.63	3.39
	4	2.85	<0.001	2.47	3.29
	5	2.88	<0.001	2.37	3.49
	6+	2.91	<0.001	2.40	3.53
**Number of emergency admissions in the previous 12 months**	0	1			
	1	0.92	<0.001	0.88	0.96
	2	0.84	<0.001	0.78	0.91
	3	0.92	0.058	0.84	1.00

## Discussion

We have shown that the recorded use of a brain scan in the management of stroke patients is increasing in English public hospitals. This may be a true result or may be due to the fact that the codes to identify brain scans in administrative data, which were only introduced in 2006, are simply better recorded and therefore reflect an increase in coding quality.

We have also shown that there is a wide variation in brain-imaging scan utilisation among English hospitals. Scans appear to be performed more often in men and the young. Moreover, there is an association with socio-economic status, with more-deprived patients having less chance of being tested in timely fashion. There are some limitations to this analysis. The results may have been chance findings although we obtained very small P values and the funnel plots have shown a greater variation than the expected due to chance (with many observations far from the 99.8% control limits). Data quality could affect the variation in rates of brain scan between trusts, if some trusts are not recording this procedure correctly. However, reimbursement is now linked to correct coding, suggesting there are likely be internal mechanisms to correct any such errors. Moreover, we restricted the inter-hospital analysis to the financial year 2008/09, the latest available, excluding the first two years of available scanning data (since the introduction of these procedure codes) in which coding is likely to have been less consistent.

Another limitation regards the definition of stroke. If hospitals define a stroke in different ways, this would decrease the robustness of our denominators. Nevertheless, it is likely that any variation in definition can only partially explain the large range described in the adjusted funnel plot.

To evaluate the extent of these limitations, the results were compared with the findings from the biennial National Sentinel Stroke Audit (NSSA) [11], which collects self-reported data from hospital sites on the first 60 consecutive cases with a primary diagnosis of stroke (I61, I63 and I64) admitted between 1 April and 30 June. The 2008 audit report showed that, in England, 57 per cent of patients were scanned within 24 hours of stroke, with 43 per cent in 2006. These NSSA results are not directly comparable with the figures reported in this paper as the NSSA data (which are based on approximately 14 per cent of all annual stroke admissions) are measuring from the onset of stroke and measure the time lapse in minutes, whereas HES measures (for all hospital patients) time from admission and by calendar day. However, it is reassuring that the NSSA 2008 figure (57 per cent) is bounded by the lower estimate (34.7 per cent scanned on same day as admission) and upper estimate (59.0 per cent scanned within one day of admission, see Table 1) from our HES-based estimate.

Whilst the National Stroke Strategy and Clinical Guidelines suggest that scanning should be immediate, the maximum resolution of HES data is one day. Whilst the measure of same-day and next-day provides a proxy for speed of scanning, the specificity and sensitivity of these measures remain uncertain.

We have highlighted how on average the 84.3% of patients admitted to English public hospitals for a stroke receive a brain scan during the hospitalisation (Table 1). According to guidelines the rate should be as close to 100% as possible. Some hospitals are very close to this value and the national average is decreased by hospitals having very low rates. In fact, given the heterogeneity of hospital rates shown in the funnel plot (Figure 3) the national average cannot be considered a good unique indicator for the performance of the English system as a whole. However, it would be interesting to compare this result with the rates in other countries. Unfortunately until now nobody has carried out in other countries the kind of study that we have carried out in England, i.e. the systematic analysis of all records of all public hospitals. Roger Beech et al. carried out an international comparison using clinical data from nine European hospitals: two in UK, one in France, one in Italy, one in Portugal, one in Spain, an three in Germany. In UK one hospital had a 29.7% rate and the other one had a 71.8%. The other hospitals ranged from 68.3% to 98.2% [12]. These results are compatible with the hypothesis that even within Europe there is a variation in brain scan performance for stroke patients.

In English NHS acute trusts, the criteria for the choice whether or not to perform a brain-imaging scan in case of stroke therefore appear unclear. Although we could not take into account all possible factors influencing the performance of BIS and therefore we could not fully elucidate the reasons for the detected heterogeneity, our results suggest that young, male, least socio-economically deprived patients, and patients with comorbidity appear to have more chances of being selected for a brain scan. Those disparities do not fully explain the reasons for the excess performance variation that we detected for two reasons. On the one hand, variables such as sex and socio-economic deprivation have shown small effects, although showing great statistical significance. On the other hand, we could not consider important variables such as structural and organisational hospital requirements. However, those disparities, especially regarding age and comorbidity, must be considered of ethical and political interest.

### Conclusion

According to clinical guidelines, every patient affected by stroke should be given a brain-imaging scan. Our analysis has shown that many healthcare providers appear not to be doing so, particularly in the elderly and those with comorbidities. There is a large variation of the use of brain-imaging for stroke patients among English NHS hospitals and some inappropriate patient selection criteria might be applied.
